# Supplementary material for: Integrated MRI–Immune–Genomic Features Enclose a Risk Stratification Model in Patients Affected by Glioblastoma
Source: Cancers (Basel). 2022 Jul 1;14(13):3249. doi: 10.3390/cancers14133249 (PMC9265092; doi:10.3390/cancers14133249)
Supplement: Supplementary file 1 [file cancers-14-03249-s001.zip › cancers-1766787-supplementary.pdf]

# Supplementary Figure S1

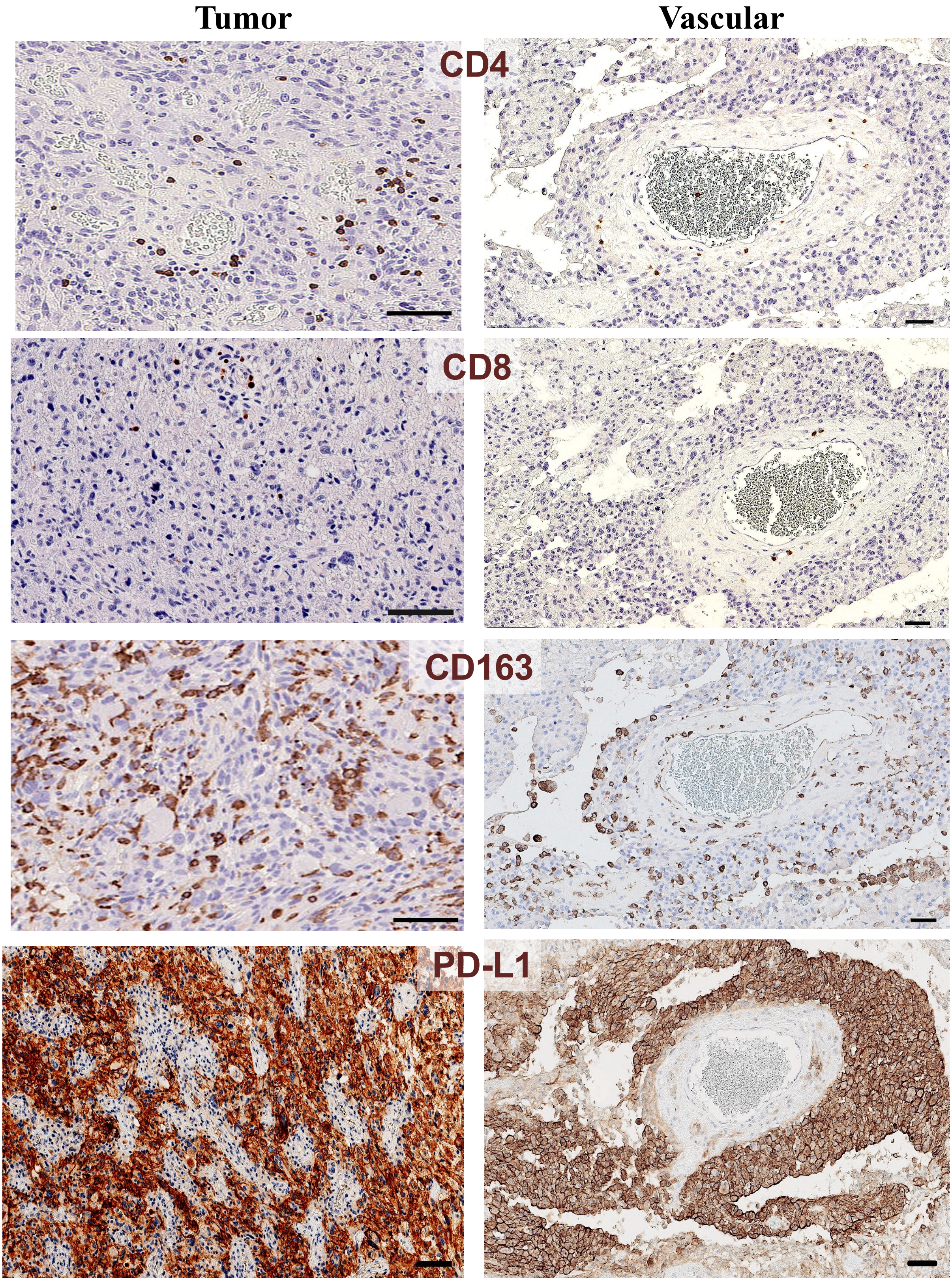


**Glioblastoma Immune Microenvironment**. Representative images of immunoperoxidase stained sections of glioblastoma to illustrate, at tumor and vascular sites, CD3+ and CD8+ Tumor Infiltrating Lymphocytes (TILs), CD163+ Tumor Associated Macrophages (TAM), and PD-L1 expression. The periarteriolar immune context (right panel) is shown on serial sections to highlight in the same microscopic field the distribution of TILs and TAMs and the strong PD-L1 expression of cancer cells insidiously surrounding the vascular wall. Scale Bars: left panel-100μm; right panel-50 μm.

# Supplementary Figure S2

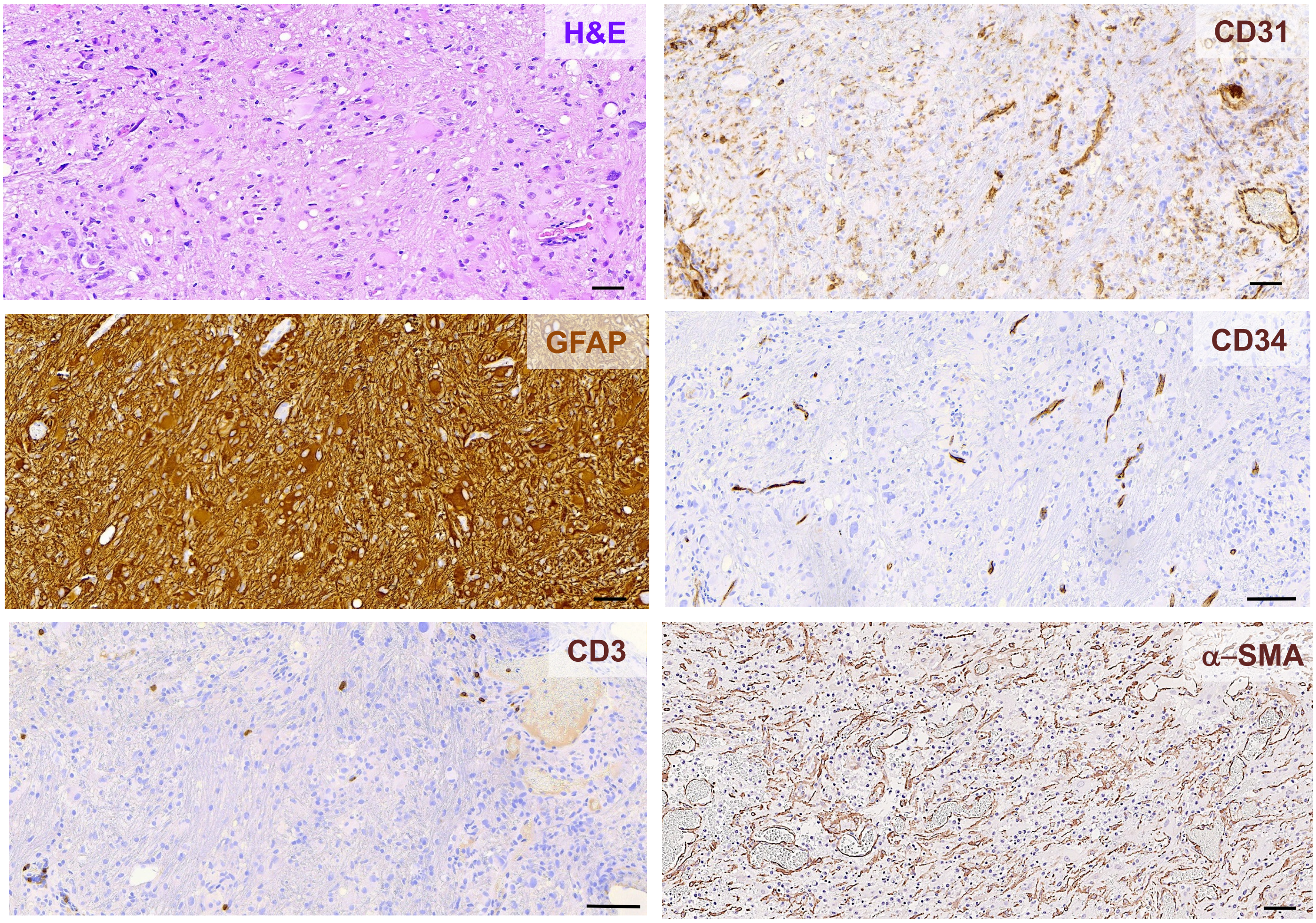


**Glioblastoma associated vascular phenotypes**. Left Panel: sections from FFPE samples of glioblastoma stained by Hematoxylin & Eosin (H&E) or immunoperoxidase for the detection of GFAP in cancer cells and CD3+ tumor infiltrating lymphocytes. Right Panel: immunoperoxidase staining of vascular profiles labeled by CD31 (PECAM-1), CD34 or alfa-Smooth Muscle Actin (a-SMA). Note the greater incidence of CD31+ vessels with respect to CD34 and the extensive α-SMA labelling, strongly suggesting an intense pericyte coverage of the tumor stroma. Scale Bars: 50um.

# Supplementary Figure S3

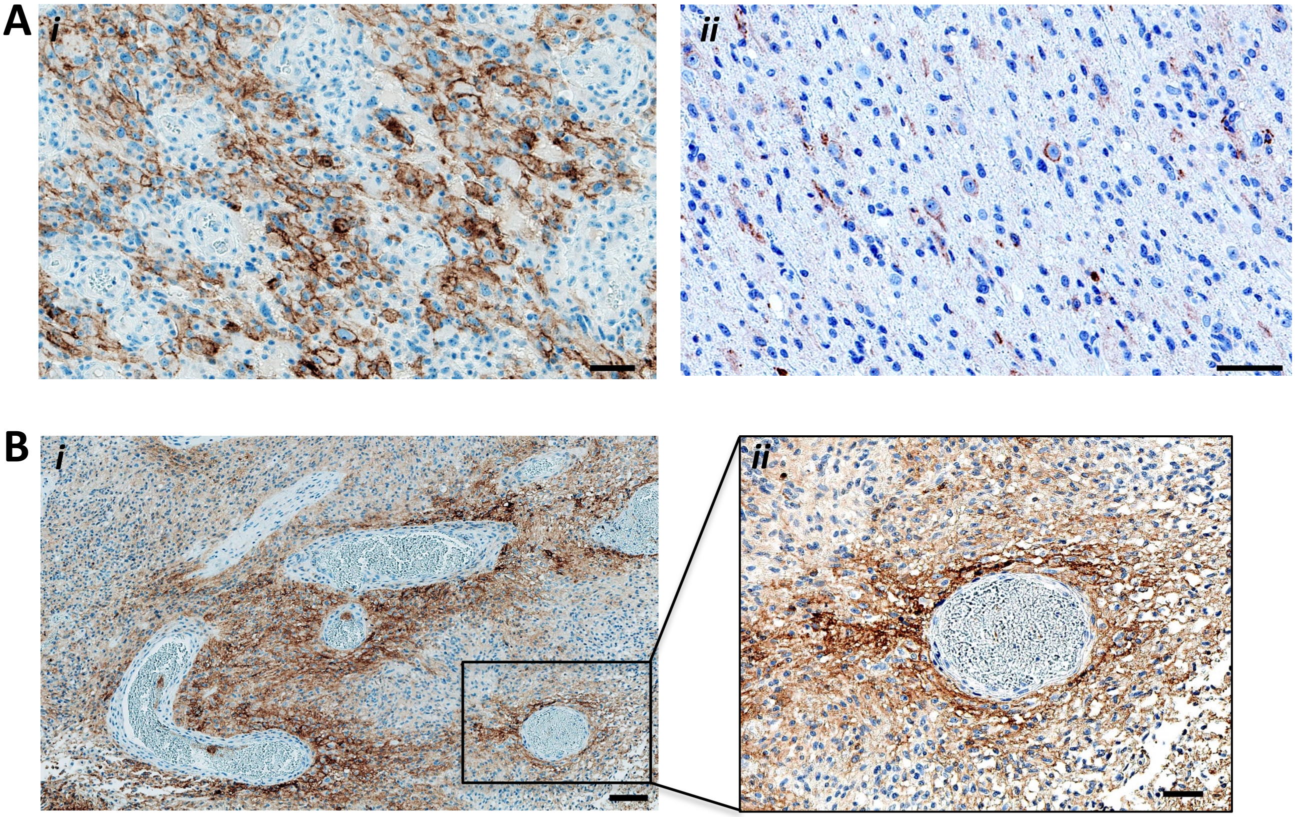


**Patterns of PD-L1 Expression. A*i*:** Immunoperoxidase stained sections from a surgically resected glioblastoma documenting the strong cytoplasmic and surface expression of PD-L1 in cancer cells. PD-L1 is also expressed by non neoplastic cells as it is occasionally detectable in neurons (**A*ii***) recognized by neurites. **B*i***: section from a case of glioblastoma showing an increasing tumor PD-L1 gradient toward arteriolar profiles. The rectangle inscribes an area shown at higher magnification in **B*ii***. Nuclei are counterstained by light hematoxylin. Scale Bars: A*i*, A*ii*, B*ii*=50 μm; B*i*=100μm.
